# Barriers to hydration and dietary sodium reduction for kidney stone prevention: a population-based study with 12-month clinical outcomes

**DOI:** 10.3389/fpubh.2025.1721782

**Published:** 2026-01-13

**Authors:** Lihan Wei, Fei Wei, Ye Wu, Yongxiang Yi

**Affiliations:** Urinary Surgery, The Third Affiliated Hospital of Zhejiang University of Traditional Chinese Medicine, Hangzhou, Zhejiang, China

**Keywords:** dietary sodium, hydration adherence, KAP, kidney stone disease, stone recurrence

## Abstract

**Background:**

The global burden of kidney stone disease is increasing, with prevention reliant on achieving high urine volume and reducing dietary sodium. However, real-world adherence and its behavioral, environmental, and occupational drivers remain poorly understood, with limited studies validating self-reported behaviors against 24-h urine biomarkers.

**Methods:**

In a single-center, clinic-based cross-sectional study with 12-month follow-up, 1,723 adults completed structured Knowledge–Attitudes–Practice questionnaires. A biomarker sub-study (*n* = 872) validated self-reported adherence against urine volume (≥2.5 L/day) and urinary sodium (≤100 mmol/day). Outcomes were adherence and symptomatic recurrence at 12 months; model performance was summarized using C-statistics and calibration indices. Multivariable logistic models identified predictors of adherence and recurrence.

**Results:**

Among 1,723 participants, adherence was 56.5% for hydration, 3.0% for sodium, and 1.2% for both; mean urine volume was 2.3 L/day and sodium excretion 178.4 mmol/day (≈10.3 g/day salt). Self-report showed high accuracy (hydration: sensitivity 87.2%, specificity 91.7%; sodium: sensitivity 78.6%, specificity 95.3%); prior counseling, higher knowledge, and greater self-efficacy increased adherence, while access/affordability barriers reduced it (C-statistics: 0.698 hydration, 0.784 sodium, 0.843 composite). Hydration adherence was lower with night shift (OR 0.67), rotating shifts (OR 0.58), limited workplace water (OR 0.52), and restricted bathrooms (OR 0.64), but higher at ambient temperature >25 °C (OR 1.67). At 12 months, recurrence was 18.1% (312/1,668); hydration (OR 0.68) and sodium adherence (OR 0.31) were protective, as were higher urine volume (OR 0.54 per L) and citrate (OR 0.93 per 100 mg/day), whereas higher urinary sodium (OR 1.42 per 50 mmol/day), calcium (OR 1.15 per 50 mg/day), and ambient temperature (OR 1.19 per 5 °C) increased risk; the recurrence model C-statistic was 0.723.

**Conclusions:**

Sodium restriction adherence is low, hydration moderate, biomarkers validate behaviors. Counseling, knowledge, self-efficacy, urban residence are associated with adherence; barriers impede it. Integrated interventions with monitoring and support may help reduce recurrence.

## 1 Introduction

Kidney stone disease (KSD) represents a growing global public health concern with substantial clinical and economic consequences. Contemporary analyses from the Global Burden of Disease project indicate that incident urolithiasis affects tens of millions worldwide annually and has continued to rise in many regions, driven by demographic aging, dietary transitions, and climate-related exposures (1). In parallel, an expanding epidemiological literature delineates a complex interplay between metabolic comorbidities, ambient temperature, and modifiable behavioral factors in KSD pathogenesis and recurrence (1, 2, 63, 64). Within this framework, hydration and dietary sodium restriction are consistently identified as first-line, low-cost preventive strategies endorsed across international guidelines, reflecting robust evidence that urine dilution and reduced urinary calcium excretion mitigate lithogenic supersaturation (3–5). Notwithstanding the apparent simplicity of these recommendations, real-world adherence remains suboptimal, thereby perpetuating avoidable morbidity and resource use (6–9). These trends underscore the need to interrogate patient-level knowledge, attitudes, and practices (KAP) that shape adherence to hydration and low-sodium advice in diverse settings.

Hydration sufficiency is central to both primary and secondary prevention of KSD. Meta-analytic data and guideline-linked reviews converge on the conclusion that achieving a daily urine volume of at least 2.0–2.5 L (typically requiring fluid intake ≥2.5 L/day) reduces incident and recurrent stone events by meaningful margins (3–5, 9, 10). Mechanistically, increased urine flow lowers ionic activity products for calcium oxalate and uric acid, thereby decreasing crystal nucleation and aggregation; these effects have been corroborated in randomized and cohort studies across risk groups (6, 8, 10). More recently, implementation-focused work has emphasized that monitoring strategies (for example, urine specific gravity/osmolality feedback) and digital behavioral tools can operationalize fluid targets, with early trials of connected-bottle, just-in-time prompting showing promise in sustaining adequate intake in recurrent stone formers (7, 9, 10). In aggregate, the evidence base positions hydration as a cornerstone of prevention while highlighting persistent behavioral and contextual barriers to consistent fluid consumption in everyday life.

In contrast, dietary sodium reduction exerts its preventive effect primarily by attenuating renal calcium excretion and, secondarily, by favorably influencing urinary citrate (4, 6, 11). Contemporary guidance from the American Urological Association (AUA) and corroborative reviews recommend limiting sodium intake to ≤2,300 mg/day (≈100 mmol/day), with stricter targets of ~1,500 mg/day reasonable for recurrent calcium stone formers with hypercalciuria (3, 5, 10). Experimental feeding studies and large cohorts demonstrate that higher sodium intake linearly increases urinary calcium and the risk of incident stones, whereas low-sodium dietary patterns decrease calciuria and lower recurrence (11, 12). Yet, achieving and maintaining sodium targets is challenging in many populations due to entrenched food environments, cultural taste preferences, and limited nutrition literacy—barriers that KAP frameworks are specifically designed to elucidate.

The Chinese context provides a salient case study for examining adherence to hydration and sodium recommendations. National ultrasound-based survey data estimate that approximately one in seventeen Chinese adults currently has KSD, with an age- and sex-adjusted prevalence of 5.8% and evidence of increasing burden over recent decades (13, 14). Furthermore, multiple time-series and case-crossover studies from China link heat exposure with acute rises in urolithiasis presentations, consistent with dehydration-mediated risk pathways; relative risks increase at higher ambient temperatures and heat stress indices, with the strongest effects observed in warmer regions and older adults (15, 16). These climatic pressures compound behavioral determinants, rendering hydration guidance both indispensable and difficult to implement during work and travel in hot seasons.

Sodium exposure in China is distinctive in both magnitude and source distribution. A comprehensive meta-analysis of 24-h urinary studies concluded that average sodium intake across Chinese age groups remains approximately double recommended maxima, with persistently higher levels in the North despite recent declines (10). Importantly, unlike many Western settings where processed foods dominate sodium exposure, contemporary dietary surveys show that a large share of sodium in Chinese adults originates from home cooking and condiments (e.g., salt, soy sauce, fermented sauces), with restaurant dishes often exceeding sodium benchmarks (17–19).

Despite the high burden of KSD in China, empirical data on the behavioral determinants of adherence to hydration and low-sodium recommendations remain sparse. While international studies document various attitudinal and environmental barriers (6, 13, 18), KAP research in China is emergent and has not deeply explored these two core preventive behaviors. A significant knowledge gap exists regarding the specific, multi-domain barriers—spanning access, cost, taste, and culinary norms—that Chinese patients face. Furthermore, few local studies have integrated crucial environmental and occupational determinants like regional climate and workplace conditions, or, critically, validated subjective patient reports against objective 24-h urine biomarkers, leaving a key discordance between patient cognition and actual behavior unexplained (20–22).

Therefore, this study aimed to characterize the KAP concerning hydration and low-sodium diets among kidney stone patients in China. We sought to identify key predictors of adherence, validate self-reported behaviors against objective 24-h urine biomarkers, and ultimately evaluate the association between these practices and 12-month stone recurrence. The overarching goal is to generate actionable evidence to inform the development of tailored and culturally resonant interventions for patients in this region.

## 2 Methods

### 2.1 Study design and setting

This investigation was designed as a single-center, clinic-based cross-sectional study with a nested prospective follow-up for clinical recurrence at 12 months and a biomarker validation sub-study using 24-h urine collections. Participants were enrolled at the Urinary Surgery (Department of Urology), The Third Affiliated Hospital of Zhejiang University of Traditional Chinese Medicine (Hangzhou, Zhejiang, China), a tertiary urology center serving predominantly urban and peri-urban populations during routine outpatient visits. All measurements and interviews were conducted at a single baseline visit in private rooms by trained research staff using a standardized protocol. Prevention targets (hydration and dietary sodium restriction) followed contemporary guideline thresholds and mechanistic evidence, and constructs were framed using a Knowledge–Attitudes–Practices (KAP) approach appropriate for behavioral determinants of kidney stone prevention (23–25).

### 2.2 Participants and eligibility

Eligible individuals were adult outpatients (≥18 years) with clinician-diagnosed urolithiasis (incident or recurrent) able to complete a structured interview. Consecutive eligible outpatients were approached over a continuous recruitment period, with all consenting individuals enrolled at the baseline visit. A total of 1,723 participants formed the analytic cohort. The biomarker sub-study enrolled all willing participants who agreed to complete a 24-h urine collection; completeness was confirmed using pre-specified criteria, including no missed voids by report, plausible total volume, and sex- and body-size-consistent creatinine excretion; samples failing these checks were excluded from validation analyses. To assess potential selection bias in the sub-study, baseline sociodemographic and clinical characteristics were systematically compared between participants who provided a valid urine sample (*n* = 872) and those who did not (*n* = 851).

Longitudinal ascertainment of symptomatic stone recurrence was performed for up to 12 months after baseline among participants with valid contact information and access to medical records; the follow-up rate was 96.8% (*n* = 1,668). All consecutive eligible patients who agreed to participate were enrolled. The lack of systematic refusal tracking introduces potential selection bias toward more motivated or health-literate participants, which may inflate adherence rates and limit generalizability to typical clinical populations.

### 2.3 Data collection procedures

Interviewer-administered questionnaires were completed in Mandarin using a standardized script and visual aids for portion sizes and fluid volumes. Interviews assessed sociodemographic characteristics, clinical history, environmental and occupational exposures, counseling history, KAP constructs, perceived barriers, and self-reported adherence behaviors. Height and weight were measured on calibrated devices with participants in light clothing and no shoes; body mass index (BMI) was calculated as kg/m^2^. The KAP questionnaire was adapted from recent, validated instruments in urolithiasis and chronic kidney disease populations and pilot-tested to ensure clarity and cultural appropriateness (22, 26). Questionnaire skip logic minimized respondent burden and ensured internal consistency.

### 2.4 Variable definitions and measurements

All variables used in analyses were defined as a priori, with coding rules applied uniformly. Higher values on all KAP and practice scales indicate more favorable beliefs or behaviors after reverse-scoring where necessary.

#### 2.4.1 Sociodemographic variables

Age was recorded in completed years and analyzed as a continuous variable. Sex was coded as male/female. Usual residence was self-reported and dichotomized as urban vs. rural according to administrative designation. Educational attainment was classified as secondary or less vs. university. Household income was self-reported, and participants were ranked into equal thirds to derive household income tertiles (low/middle/high) within the study population.

#### 2.4.2 Clinical variables

BMI (kg/m^2^) was analyzed continuously. Physician-diagnosed comorbidities were captured via structured checklist and coded as binary indicators (hypertension; diabetes; gout/hyperuricemia). In addition, a composite of any comorbidity indicator was defined (1 if ≥1 of hypertension, diabetes, or gout/hyperuricemia; otherwise, 0). Stone history included a count of previous symptomatic episodes confirmed by history and records (numeric variable). For subgroup analyses, stone history was also dichotomized as first episode (index stone event only) vs. recurrent (≥2 documented episodes including the current presentation). “Prior counseling” was defined as documentation or self-report of having received structured advice from a clinician or dietitian regarding hydration and/or dietary sodium restriction before the baseline visit (yes/no).

#### 2.4.3 Knowledge constructs

Knowledge items were derived from guideline-concordant content and key risk mechanisms. Each correct response scored 1 point; incorrect/“don't know” scored 0. The overall knowledge score (0–10) summed all items (Cronbach's α = 0.82). Subscales included hydration-guideline knowledge (e.g., fluid/urine volume targets; 0–3; α = 0.76), sodium-guideline knowledge (e.g., daily sodium limits and sources; 0–3; α = 0.79), and risk-factor awareness (e.g., heat exposure, obesity, metabolic abnormalities; 0–4; α = 0.74). Item pools and scaling were adapted from recent KAP questionnaires with demonstrated reliability in stone-forming and CKD populations (22, 26). Scores were analyzed per point.

#### 2.4.4 Attitudes (health belief model constructs)

Attitudes were measured using 5-point Likert items (1 = strongly disagree to 5 = strongly agree), averaged within domains: perceived susceptibility (α = 0.71), perceived severity (α = 0.69), perceived benefits of hydration/sodium restriction (α = 0.77), and self-efficacy for performing these behaviors across routine contexts (α = 0.84). Domain definitions and items mirrored formats used in recent KAP applications to urolithiasis prevention (22, 26–28).

#### 2.4.5 Practices/behaviors

Behavioral items used 5-point frequency scales, though the validity of self-reported dietary and fluid intake behaviors against objective measures remain uncertain beyond the subset with 24-h urine validation. Recalling bias and social desirability bias may influence these self-reported measures. Fluid intake (workday and free day) was recorded in liters/day based on participant report of typical consumption; values were analyzed continuously and used in adherence definitions. Water-bottle carrying frequency was assessed on a 5-point scale (1 = never to 5 = always; α = 0.73). Sodium-related practices included added-salt use at the table/cooking (reverse-scored so higher values reflect less discretionary salt), processed-food avoidance (α = 0.76), and nutrition-label reading frequency (α = 0.78). Processed food consumption was recorded as the number of meals per week containing processed or packaged foods typically high in sodium (e.g., instant noodles, cured meats, pickled products, canned goods, savory snacks); this variable was used to operationalize the adherence threshold of ≤2 meals/week. Eating-out frequency was captured categorically as ≤1 time/week, 2–3 times/week, or >3 times/week and used as a social-environment covariate.

#### 2.4.6 Perceived barrier domains

Perceived barriers to adherence were assessed with 5-point Likert items (1 = strongly disagree to 5 = strongly agree) and averaged within domains pre-specified a priori: taste/palatability, environmental/heat, health-care system/logistics, access/availability, information, and affordability. Higher scores indicate greater barrier burden. Domain scores were analyzed as continuous variables.

#### 2.4.7 Occupational and environmental exposures

Work shift pattern was categorized as day shift (07:00–19:00; reference), night shift (19:00–07:00), or rotating shifts. Physical work intensity was recorded as sedentary, moderate, or heavy based on job demands. Workplace water availability (always/usually/limited or never) and bathroom access (unrestricted/restricted) were assessed by single items referencing the participant's current job. Family support for dietary changes was classified as high, moderate, or low/none via a single global item. Water quality at home was self-rated as excellent/good vs. fair/poor based on taste, odor, clarity, and perceived safety. Distance to healthcare facility was self-reported as typical door-to-door travel time and dichotomized as <30 vs. ≥30 min. Household cooking responsibility was categorized as self/spouse primary cook vs. others (other family members, cafeteria, takeaway). Ambient temperature exposure was assigned at the city level for the month preceding interview and categorized a priori as <15 °C (reference), 15–25 °C, and >25 °C; temperature was also analyzed continuously (per 5 °C increase) in sensitivity analyses (24).

### 2.5 Primary and secondary outcomes

#### 2.5.1 Adherence outcome definitions

The primary outcomes were adherence to hydration and sodium prevention targets at baseline, operationalized from self-reported behaviors, with a secondary composite outcome requiring both targets.

1. The 2.5 L/day fluid intake threshold derives from meta-analyses demonstrating that achieving urine volumes ≥2.0 L/day reduces stone recurrence by 50–60%. Water-bottle carrying frequency (≥4/5 scale, “often” or “always”) was selected as a behavioral proxy for consistent hydration effort; this threshold lacks formal validation but reflects high-frequency positive behaviors commonly employed in KAP research. For sodium adherence, experimental feeding studies demonstrate that each 100 mmol/day increment in urinary sodium increases urinary calcium excretion and stone risk. The processed-food threshold (≤2 meals/week) was based on dietary survey data indicating that single processed meals in China (instant noodles, restaurant dishes, cured meats) typically contain 1,500–2,500 mg sodium, meaning >2 such meals weekly likely preclude achieving daily sodium limits. These composite criteria require formal validation through repeated 24-h urine measurements (29– 32).
2. Sodium adherence (primary) was defined as reporting low-sodium dietary practice “often/always” (≥4 on a 5-point scale) and processed-food consumption ≤2 meals/week.
3. Composite adherence (secondary) required simultaneous fulfillment of the hydration and sodium definitions.

#### 2.5.2 Biomarker validation

A secondary objective validated these self-report outcomes against 24-h urine biomarkers in the sub-study (*n* = 872): urine volume (L/day) and urine sodium excretion (mmol/day). Diagnostic performance of the self-report definitions was quantified against biomarker thresholds of urine volume ≥2.5 L/day (hydration) and urine sodium ≤100 mmol/day (sodium), consistent with prevention targets and the use of 24-h urinary sodium as a biomarker of dietary sodium intake (23–25, 33, 34).

#### 2.5.3 Stone recurrence outcome

Clinical recurrence over 12 months after baseline was defined as a symptomatic stone event requiring medical attention and/or imaging confirmation documented in the electronic medical record or verified via structured telephone follow-up with the participant. Date of recurrence, care setting, and management (e.g., analgesia, expulsive therapy, surgery) were abstracted when available. Participants without events were classified as non-events at their last contact within the 12-month window.

### 2.6 24-h urine collection and laboratory assays

Participants in the biomarker sub-study received written and verbal instructions for single 24-h urine collection beginning after discarding the first morning void and including all subsequent voids up to and including the first void the following morning. Collections were refrigerated during the collection period and transported to the hospital laboratory within 4 h of completion. Total urine volume was measured gravimetrically assuming a density of 1.0 kg/L. Urine sodium (mmol/day) was quantified on automated analyzers; calcium and uric acid were measured enzymatically, citrate by enzymatic/colorimetric assay, and oxalate by enzymatic assay according to manufacturer protocols. Urine osmolality was determined by freezing-point depression osmometry and urine specific gravity by refractometry. Internal quality control procedures followed laboratory standards. All 872 participants in the biomarker sub-study provided complete collections, and results were linked to questionnaire data by unique identifiers.

### 2.7 Statistical analysis

Analyses followed a pre-specified plan. Continuous variables were summarized as mean (SD) or median (IQR) and categorical variables as number (percentage). Group differences in KAP scores were assessed using independent-samples *t* tests (two groups) or one-way ANOVA (≥3 groups), with Welch corrections when homoscedasticity was violated. Primary outcomes—hydration adherence, sodium adherence, and composite adherence—were modeled with separate multivariable logistic regressions to obtain adjusted odds ratios (ORs) and 95% confidence intervals (CIs). Covariates were age, sex, residence (urban/rural), education, income tertile, BMI, previous stone episodes, any comorbidity, prior counseling, the overall knowledge score, attitude domains (susceptibility, severity, perceived benefits, self-efficacy), and barrier domains (access, affordability, information, environmental). The sodium adherence model (51 events, 14+ predictors; events-per-variable [EPV] ratio ≈3–4:1) falls below recommended 10–15:1 thresholds, increasing overfitting risk; this model is exploratory and requires validation in larger samples. Model performance was assessed with the C-statistic (bootstrap 95% CIs), Nagelkerke *R*^2^, and the Hosmer–Lemeshow goodness-of-fit test.

Twelve-month stone recurrence was analyzed with multivariable logistic regression using the same covariates; biomarker-augmented models additionally included 24-h urine volume, sodium, calcium, and citrate. For significant predictors in the fully adjusted recurrence model, population attributable fractions (PAFs) and 95% CIs were computed. Construct validity was examined with Pearson correlations (Fisher's z-transformed 95% CIs). A pre-specified structural equation model (knowledge → attitudes → behaviors → adherence), adjusted for the covariates above, was fit; we report Comparative Fit Index (CFI), Root Mean Square Error of Approximation (RMSEA), Standardized Root Mean Residual (SRMR), indirect effects from bootstrap resampling, and outcome-specific *R*^2^ values.

Environmental and occupational factors were evaluated in covariate-adjusted logistic models; effect modification by residence (urban vs. rural) was tested with factor × residence interactions and likelihood-ratio tests. Sodium-related models additionally adjusted for family support, household cooking responsibility, eating-out frequency, home water quality, and travel time to care. Missing data were handled under a missing-at-random assumption using multiple imputation by chained equations including all analysis variables and outcomes; pooled estimates were obtained using Rubin's rules. The structural equation model was fit using a robust estimator, with standardized path coefficients reported; indirect effects were quantified via bias-corrected bootstrap. Model adequacy was evaluated with CFI, RMSEA, and SRMR. Sensitivity analyses repeated key models in complete cases and in the biomarker subset. All tests were two-sided at α = 0.05 without multiplicity adjustment given the exploratory nature of environmental analyses. Results are presented as ORs with 95% CIs and supporting diagnostics.

## 3 Results

### 3.1 Participant characteristics and baseline adherence

Among 1,723 participants [mean age 52.3 years [SD 12.7]; 65.4% male; 60.3% urban], the median prior stone episode count was 2 (IQR 1–4), and 39.6% had received prior dietary counseling (Table 1). Comorbidities included hypertension (30.2%), diabetes (10.9%), and gout or hyperuricemia (14.2%). Mean knowledge score was 6.0 of 10 (SD 2.1), while perceived severity of stone disease was high (4.1 of 5, SD 0.9) and self-efficacy moderate (3.4 of 5, SD 1.2).

**Table 1:** Baseline characteristics of study participants.

**Characteristic**	**Overall (*N* = 1,723)**
**Demographics**	
Age, mean (SD), y	52.3 (12.7)
Age, median (IQR), y	51.0 (43-60)
**Sex, no. (%)**	
Male	1,126 (65.4)
Female	597 (34.6)
**Residence, no. (%)**	
Urban	1,039 (60.3)
Rural	684 (39.7)
**Education, no. (%)**	
Secondary or less	999 (58.0)
University	724 (42.0)
**Clinical characteristics**	
BMI, mean (SD), kg/m^2^	24.8 (3.9)
**Comorbidities, no. (%)**	
Hypertension	521 (30.2)
Diabetes	187 (10.9)
Gout/hyperuricemia	245 (14.2)
Stone episodes, median (IQR)	2 (1–4)
Prior counseling received, no. (%)	683 (39.6)
**Knowledge, attitudes, and practices**	
Knowledge score (0–10), mean (SD)	6.0 (2.1)
Perceived susceptibility (1–5), mean (SD)	3.2 (1.1)
Perceived severity (1–5), mean (SD)	4.1 (0.9)
Perceived benefits (1–5), mean (SD)	3.8 (1.0)
Self-efficacy (1–5), mean (SD)	3.4 (1.2)
**Primary outcomes**	
Hydration adherent, no. (%)	974 (56.5)
Sodium adherent, no. (%)	51 (3.0)
Composite adherent, no. (%)	20 (1.2)
Stone recurrence at 12 months, no. (%)	312 (18.1)
**24-h urine parameters** ^a^	
Urine volume, mean (SD), L	2.3 (0.8)
Urine sodium, mean (SD), mmol/d	178.4 (76.2)
Urine calcium, mean (SD), mg/d	185.4 (89.7)
Urine citrate, mean (SD), mg/d	524.6 (287.9)

BMI, body mass index; IQR, interquartile range.

^a^24-h urine data were available for 872 participants (50.6%).

**Figure 1:**
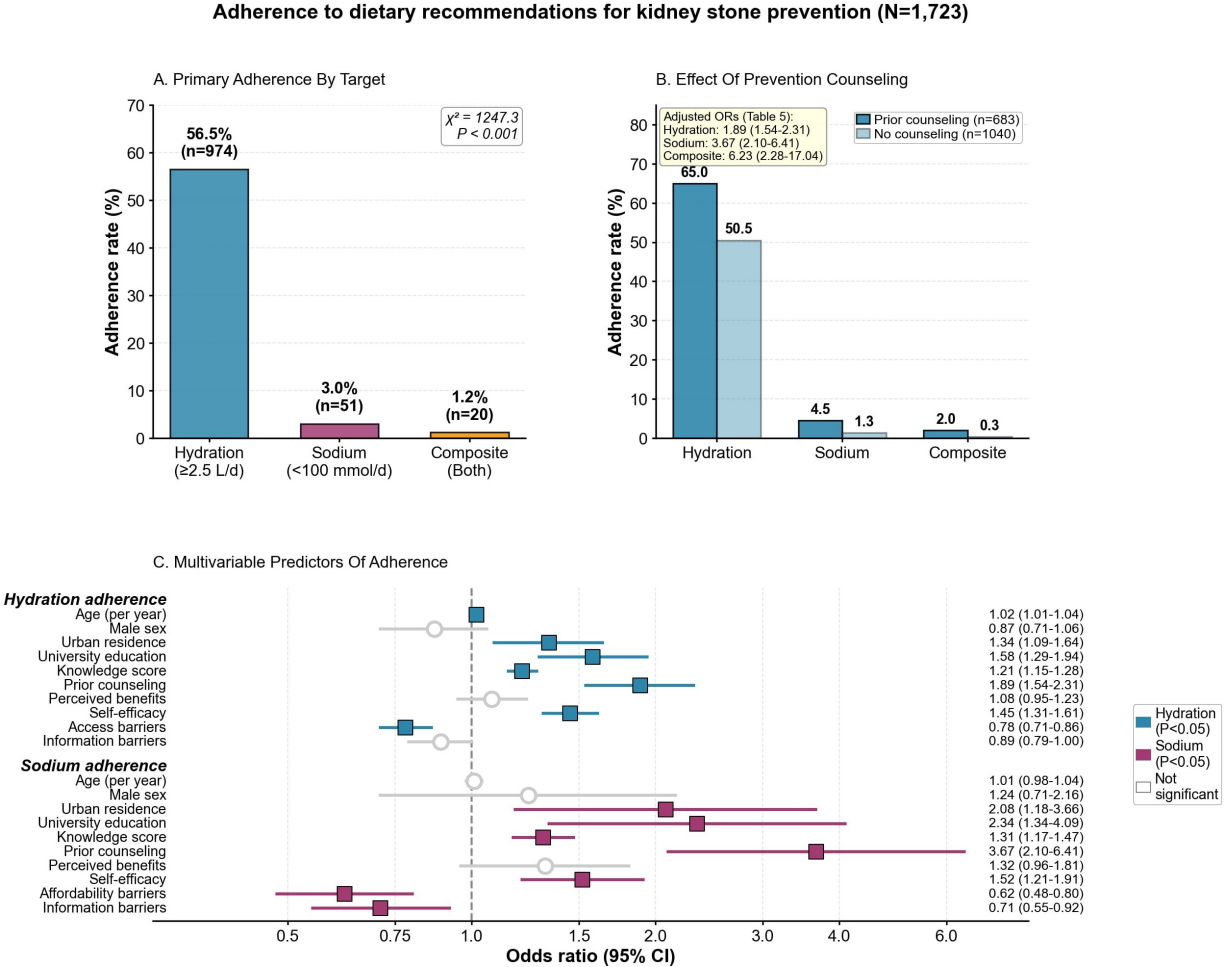
Adherence to guideline-concordant prevention behaviors in nephrolithiasis. **(A)** Baseline prevalence of adherence by target (hydration, sodium restriction, and their composite), using pre-specified questionnaire-derived operational definitions detailed in the Methods. **(B)** Adherence stratified by receipt of prior prevention counseling, illustrating unadjusted differences between exposed and unexposed groups. **(C)** Determinants of adherence from multivariable logistic regression, displayed as adjusted odds ratios with 95% confidence intervals; models are covariate-adjusted for demographic and clinical factors, educational attainment, urbanicity, prior counseling, Knowledge–Attitudes–Practice and Health Belief Model constructs, and predefined barrier domains. OR, odds ratio; CI, confidence interval.

Baseline adherence rates were 56.5% for hydration (*n* = 974), 3.0% for sodium (*n* = 51), and 1.2% for composite targets (*n* = 20; Figure 1A). Among 872 participants (50.6%) providing 24-h urine collections, mean volume was 2.3 L/day (SD 0.8) and mean sodium excretion 178.4 mmol/day (SD 76.2), corresponding to approximately 10.3 g/day dietary sodium intake. Biomarker participants vs. non-participants showed no significant differences in mean age (52.1 ± 12.5 vs. 52.5 ± 12.9 years, *P* = 0.42), male sex proportion (66.2% vs. 64.5%, *P* = 0.38), or median prior stone episodes [2 [IQR 1-4] vs. 2 [IQR 1–4], *P* = 0.51], reducing concern for demographic selection bias. However, selection based on unmeasured behavioral or motivational factors remains possible. Adherence was consistently higher among participants who had received prior prevention counseling compared with those without counseling—hydration 65.0% vs. 50.5%, sodium 4.5% vs. 1.3%, and composite 2.0% vs. 0.3% (Figure 1B).

### 3.2 Biomarker validation

Self-reported hydration adherence correlated strongly with urine volume (Table 2). Adherent participants had mean urine volumes of 2.8 vs. 1.6 L/day in non-adherent participants (difference 1.2 L, 95% CI 1.1–1.3, *P* < 0.001, Cohen's d = 1.71), with sensitivity 87.2% (95% CI 84.1–90.3%) and specificity 91.7% (95% CI 88.5–94.9%) against the ≥2.5 L/day threshold. Sodium-adherent participants excreted 98.5 vs. 185.7 mmol/day (difference −87.2 mmol/day, 95% CI −112.4 to −62.0, *P* < 0.001, d = 1.34), with sensitivity 78.6% (95% CI 59.0–98.2%) and specificity 95.3% (95% CI 93.8–96.8%) against ≤100 mmol/day. Single-collection day-to-day variability may introduce misclassification affecting these diagnostic accuracy estimates. Knowledge scores correlated with urine volume (*r* = 0.34, *P* < 0.001) and self-efficacy inversely with urine sodium (*r* = −0.28, *P* < 0.001).

**Table 2:** Twenty-four-hours urine parameters by self-reported adherence categories.

**Biomarker**	**Adherent**	**Non-adherent**	**Difference**	***P*-value**	**Effect size^a^**
	**Mean (SD)**	**Mean (SD)**	**(95% CI)**		**(Cohen's d)**
**Hydration adherence validation (*****n*** = **872)**^b^					
24-h urine volume, L	2.8 (0.8)	1.6 (0.6)	1.2	<0.001	1.71
	(*n* = 485)	(*n* = 387)	(1.1–1.3)		
Urine osmolality, mOsm/kg	421 (156)	687 (198)	−266	<0.001	1.52
			(−298 to −234)		
Urine specific gravity	1.012 (0.008)	1.021 (0.011)	−0.009	<0.001	0.94
			(−0.010 to −0.008)		
**Sodium adherence validation (*****n*** = **872)**^c^					
24-h urine sodium, mmol/d	98.5 (45.2)	185.7 (78.3)	−87.2	<0.001	1.34
	(*n* = 28)	(*n* = 844)	(−112.4 to −62.0)		
24-h urine sodium, g/d	2.3 (1.0)	4.3 (1.8)	−2	<0.001	1.34
			(−2.6 to −1.4)		
Estimated sodium intake, g/d^d^	5.8 (2.5)	10.8 (4.5)	−5	<0.001	1.34
			(−6.5 to −3.5)		
**Additional urine parameters**					
24-h urine calcium, mg/d	172.4 (82.1)	194.8 (95.2)	−22.4	0.007	0.25
			(−38.6 to −6.2)		
24-h urine citrate, mg/d	589.3 (318.7)	478.2 (254.1)	111.1	<0.001	0.39
			(67.8–154.4)		
24-h urine oxalate, mg/d	32.8 (18.4)	38.1 (21.7)	−5.3	0.008	0.27
			(−9.2 to −1.4)		
24-h urine uric acid, mg/d	628 (287)	679 (314)	−51	0.118	0.17
			(−115 to 13)		
**Diagnostic performance**					
Hydration adherence (urine volume ≥2.5 L)	Sensitivity: 87.2% (95% CI, 84.1%−90.3%)		Specificity: 91.7% (95% CI, 88.5%−94.9%)		
Sodium adherence (urine sodium ≤100 mmol/d)	Sensitivity: 78.6% (95% CI, 59.0%−98.2%)		Specificity: 95.3% (95% CI, 93.8%−96.8%)		
**Correlation analysis** ^e^					
Knowledge score vs. urine volume	*r* = 0.34 (95% CI, 0.28–0.40)		*P* < 0.001		
Self-efficacy vs. urine sodium	*r* = −0.28 (95% CI, −0.34 to −0.22)		*P* < 0.001		

^a^>Effect size interpreted as small (0.2), medium (0.5), or large (0.8) according to Cohen's conventions.

^b^Hydration adherence defined as self-reported fluid intake ≥2.5 L/day and carrying water bottle ≥4 on 5-point scale.

^c^Sodium adherence defined as self-reported low-sodium diet adherence ≥4 on 5-point scale and processed food intake ≤2 meals/week.

^d^Estimated dietary sodium intake calculated as urine sodium × 2.5 (accounting for non-renal losses).

^e^Pearson correlation coefficients with 95% confidence intervals calculated using Fisher's z-transformation. Twenty-four-hour urine collection completed by 872 participants (50.6% of study sample). All *P*-values are 2-sided.

### 3.3 Knowledge, attitudes, and barriers

Urban residents scored higher than rural residents on knowledge (6.3 vs. 5.5, *P* = 0.003), perceived susceptibility (3.3 vs. 3.0, *P* = 0.018), perceived benefits (3.9 vs. 3.6, *P* = 0.021), and self-efficacy (3.6 vs. 3.1, *P* = 0.001; Table 3). University-educated participants scored higher across all domains (*P* < 0.001). Prior counseling recipients showed markedly elevated knowledge (6.8 vs. 5.5), perceived susceptibility (3.6 vs. 2.9), perceived benefits (4.2 vs. 3.5), and self-efficacy (3.8 vs. 3.1; all *P* < 0.001).

**Table 3:** Knowledge, attitude, and practice scores by demographic characteristics.

**Characteristic**	**Knowledge score**		**Perceived susceptibility**		**Perceived benefits**		**Self-efficacy**	
	**(0–10 scale)**		**(1–5 scale)**		**(1–5 scale)**		**(1–5 scale)**	
	**Mean (SD)**	***P*** **-value**	**Mean (SD)**	***P*** **-value**	**Mean (SD)**	***P*** **-value**	**Mean (SD)**	***P*** **-value**
Overall (*N* = 1,723)	6.0 (2.1)	–	3.2 (1.1)	–	3.8 (1.0)	–	3.4 (1.2)	–
**Sex**								
Male (*n* = 1,126)	5.9 (2.1)	0.042	3.1 (1.1)	0.087	3.7 (1.0)	0.156	3.3 (1.2)	0.024
Female (*n* = 597)	6.2 (2.0)		3.3 (1.0)		3.9 (1.0)		3.6 (1.1)	
**Residence**								
Urban (*n* = 1,039)	6.3 (2.0)	0.003	3.3 (1.0)	0.018	3.9 (0.9)	0.021	3.6 (1.1)	0.001
Rural (*n* = 684)	5.5 (2.2)		3.0 (1.2)		3.6 (1.1)		3.1 (1.3)	
**Education**								
Secondary or less (*n* = 999)	5.4 (2.3)	<0.001	3.0 (1.2)	0.002	3.6 (1.1)	<0.001	3.1 (1.3)	<0.001
University (*n* = 724)	6.8 (1.7)		3.5 (0.9)		4.1 (0.8)		3.8 (1.0)	
**Income tertile** ^a^								
Low (*n* = 574)	5.6 (2.3)	0.008	2.9 (1.2)	0.012	3.5 (1.1)	0.003	3.1 (1.3)	0.001
Middle (*n* = 575)	6.1 (2.0)		3.2 (1.0)		3.8 (0.9)		3.5 (1.1)	
High (*n* = 574)	6.3 (1.9)		3.5 (1.0)		4.0 (0.9)		3.7 (1.1)	
**Prior counseling**								
Yes (*n* = 683)	6.8 (1.8)	<0.001	3.6 (0.9)	<0.001	4.2 (0.8)	<0.001	3.8 (1.0)	<0.001
No (*n* = 1,040)	5.5 (2.2)		2.9 (1.1)		3.5 (1.0)		3.1 (1.2)	
***Stone History***								
First episode (*n* = 687)	5.7 (2.2)	0.034	2.8 (1.2)	<0.001	3.6 (1.1)	0.018	3.2 (1.2)	0.089
Recurrent (*n* = 1,036)	6.2 (2.0)		3.4 (1.0)		3.9 (0.9)		3.5 (1.1)	

^a^Income tertiles based on relative household income within the study population. *P*-values calculated using independent samples *t* tests for 2-group comparisons and ANOVA for 3-group comparisons.

Taste barriers were most prevalent (58.0% high barrier; mean 3.4, SD 1.2), followed by environmental (47.0%; 3.1, SD 1.2), healthcare system (41.0%; 2.9, SD 1.3), access (34.0%; 2.8, SD 1.3), information (31.0%; 2.6, SD 1.1), and affordability barriers (23.0%; 2.1, SD 1.2; Table 4). Rural participants and those with lower education reported higher barriers across all domains.

**Table 4:** Perceived barriers to kidney stone prevention adherence by patient characteristics.

**Barrier domain**	**Overall**	**Urban vs. rural**		**Education level**		**High barrier^a^**
	**Mean (SD)**	**Urban**	**Rural**	**Secondary**	**University**	**No. (%)**
		**Mean (SD)**	**Mean (SD)**	**Mean (SD)**	**Mean (SD)**	
Access barriers^b^	2.8 (1.3)	2.6 (1.2)	3.1 (1.4)	3.0 (1.4)	2.5 (1.2)	586 (34.0)
Water access at work	2.9 (1.4)	2.7 (1.3)	3.2 (1.5)	3.1 (1.5)	2.6 (1.3)	–
Bathroom availability	2.7 (1.3)	2.5 (1.2)	3.0 (1.4)	2.9 (1.4)	2.4 (1.1)	–
Affordability barriers^c^	2.1 (1.2)	1.9 (1.1)	2.4 (1.3)	2.3 (1.3)	1.8 (1.0)	396 (23.0)
Cost of bottled water	2.2 (1.3)	2.0 (1.2)	2.5 (1.4)	2.4 (1.4)	1.9 (1.1)	–
Fresh food costs	2.0 (1.2)	1.8 (1.1)	2.3 (1.3)	2.2 (1.3)	1.7 (1.0)	–
Taste barriers^d^	3.4 (1.2)	3.3 (1.1)	3.6 (1.3)	3.5 (1.3)	3.3 (1.1)	999 (58.0)
Plain water taste	3.5 (1.3)	3.4 (1.2)	3.7 (1.4)	3.6 (1.4)	3.4 (1.2)	–
Low-sodium food taste	3.3 (1.2)	3.2 (1.1)	3.5 (1.3)	3.4 (1.3)	3.2 (1.1)	–
Information barriers^e^	2.6 (1.1)	2.4 (1.0)	2.9 (1.2)	2.8 (1.2)	2.3 (0.9)	534 (31.0)
Understanding guidelines	2.7 (1.2)	2.5 (1.1)	3.0 (1.3)	2.9 (1.3)	2.4 (1.0)	–
Conflicting advice	2.5 (1.1)	2.3 (1.0)	2.8 (1.2)	2.7 (1.2)	2.2 (0.9)	–
Healthcare system barriers^f^	2.9 (1.3)	2.7 (1.2)	3.2 (1.4)	3.1 (1.4)	2.6 (1.1)	706 (41.0)
Provider support	3.0 (1.4)	2.8 (1.3)	3.3 (1.5)	3.2 (1.5)	2.7 (1.2)	–
Follow-up availability	2.8 (1.3)	2.6 (1.2)	3.1 (1.4)	3.0 (1.4)	2.5 (1.1)	–
Environmental barriers^g^	3.1 (1.2)	2.9 (1.1)	3.4 (1.3)	3.3 (1.3)	2.8 (1.0)	810 (47.0)
Work environment	3.2 (1.3)	3.0 (1.2)	3.5 (1.4)	3.4 (1.4)	2.9 (1.1)	–
Social pressure	3.0 (1.2)	2.8 (1.1)	3.3 (1.3)	3.2 (1.3)	2.7 (1.0)	–

^a^High barrier defined as mean score ≥4 on 1–5 scale (1 = no barrier, 5 = major barrier).

^b^Access barriers: difficulty accessing water or bathroom facilities. *P* < 0.001 for urban vs. rural and education comparisons.

^c^Affordability barriers: financial constraints on recommended foods/beverages. *P* < 0.001 for urban vs. rural and education comparisons.

^d^Taste barriers: palatability concerns with recommended dietary changes. *P* = 0.023 for urban vs. rural, *P* = 0.089 for education.

^e^Information barriers: confusion or lack of clarity about prevention guidelines. *P* < 0.001 for urban vs. rural and education comparisons.

^f^Healthcare system barriers: inadequate provider support or follow-up. *P* < 0.001 for urban vs. rural and education comparisons.

^g^Environmental barriers: work/social environment constraints. *P* < 0.001 for urban vs. rural and education comparisons.

### 3.4 Predictors of adherence

In multivariable models (Figure 1C, Table 5), prior counseling was the strongest predictor across outcomes (hydration OR 1.89, 95% CI 1.54–2.31; sodium OR 3.67, 95% CI 2.10–6.41; composite OR 6.23, 95% CI 2.28–17.04; all *P* < 0 .001). Knowledge (ORs 1.21–1.47 per point) and self-efficacy (ORs 1.45–1.89 per point) independently predicted adherence (all *P* < 0.001). Urban residence (hydration OR 1.34, 95% CI 1.09–1.64; sodium OR 2.08, 95% CI 1.18–3.66) and university education (ORs 1.58–4.58) were protective. Access barriers reduced hydration (OR 0.78, 95% CI 0.71–0.86) and composite adherence (OR 0.51, 95% CI 0.33–0.78), while affordability barriers reduced sodium (OR 0.62, 95% CI 0.48–0.80) and composite adherence (OR 0.48, 95% CI 0.31–0.74; all *P* ≤ 0.002). Model C-statistics were 0.698 (hydration), 0.784 (sodium), and 0.843 (composite).

**Table 5:** Multivariable logistic regression models for adherence behaviors.

**Predictor variable**	**Hydration adherence**		**Sodium adherence**		**Composite adherence**	
	**OR (95% CI)**	***P*** **-value**	**OR (95% CI)**	***P*** **-value**	**OR (95% CI)**	***P*** **-value**
**Demographics**						
Age (per year)	1.02 (1.01–1.04)	0.003	1.01 (0.98–1.04)	0.612	1.00 (0.95–1.05)	0.891
Male sex	0.87 (0.71–1.06)	0.168	1.24 (0.71–2.16)	0.451	1.87 (0.69–5.08)	0.219
Urban residence	1.34 (1.09–1.64)	0.005	2.08 (1.18–3.66)	0.011	3.42 (1.26–9.28)	0.016
University education	1.58 (1.29–1.94)	<0.001	2.34 (1.34–4.09)	0.003	4.58 (1.68–12.49)	0.003
High income tertile	1.21 (0.96–1.52)	0.107	1.89 (1.02–3.50)	0.043	2.84 (0.93–8.67)	0.067
**Clinical factors**						
BMI (per kg/m^2^)	0.98 (0.95–1.01)	0.189	0.95 (0.88–1.03)	0.223	0.92 (0.80–1.06)	0.256
Previous stone episodes (per episode)	1.08 (1.02–1.14)	0.007	1.12 (0.96–1.31)	0.142	1.18 (0.87–1.60)	0.287
Any comorbidity	1.23 (0.99–1.52)	0.058	1.67 (0.93–3.01)	0.087	2.15 (0.74–6.24)	0.159
Prior counseling received	1.89 (1.54–2.31)	<0.001	3.67 (2.10–6.41)	<0.001	6.23 (2.28–17.04)	<0.001
**Knowledge and attitudes**						
Knowledge score (per point)	1.21 (1.15–1.28)	<0.001	1.31 (1.17–1.47)	<0.001	1.47 (1.22–1.77)	<0.001
Perceived susceptibility (per point)	1.14 (1.01–1.28)	0.032	1.25 (0.93–1.68)	0.138	1.56 (0.92–2.64)	0.098
Perceived benefits (per point)	1.08 (0.95–1.23)	0.245	1.32 (0.96–1.81)	0.089	1.45 (0.81–2.59)	0.212
Self-efficacy (per point)	1.45 (1.31–1.61)	<0.001	1.52 (1.21–1.91)	<0.001	1.89 (1.34–2.67)	<0.001
**Perceived barriers**						
Access barriers (per point)	0.78 (0.71–0.86)	<0.001	0.84 (0.68–1.04)	0.113	0.51 (0.33–0.78)	0.002
Affordability barriers (per point)	0.91 (0.82–1.01)	0.078	0.62 (0.48–0.80)	<0.001	0.48 (0.31–0.74)	<0.001
Information barriers (per point)	0.89 (0.79–1.00)	0.053	0.71 (0.55–0.92)	0.009	0.65 (0.42–1.01)	0.056
Environmental barriers (per point)	0.82 (0.74–0.91)	<0.001	0.78 (0.62–0.98)	0.032	0.69 (0.46–1.04)	0.074
**Model performance**						
Nagelkerke *R*^2^	0.187	–	0.241	–	0.334	–
C-statistic (95% CI)	0.698	–	0.784	–	0.843	–
	(0.673–0.723)		(0.734–0.834)		(0.783–0.903)	
Hosmer–Lemeshow *P*-value	0.324	–	0.187	–	0.412	–

### 3.5 Environmental and occupational factors

Night-shift (OR 0.67, 95% CI 0.51–0.88) and rotating-shift workers (OR 0.58, 95% CI 0.44–0.76) showed reduced hydration adherence compared with day-shift workers (both *P* ≤ 0 .004), with urban-rural interaction (*P* = 0.034; Table 6). Limited workplace water availability (OR 0.52, 95% CI 0.40–0.68) and restricted bathroom access (OR 0.64, 95% CI 0.52–0.79) reduced hydration adherence (both *P* < 0.001), while heavy physical work increased both hydration (OR 1.89, 95% CI 1.44–2.48) and sodium adherence (OR 2.14, 95% CI 1.15–3.98; both *P* ≤ 0.016). Lack of primary cooking responsibility (OR 0.31, 95% CI 0.11–0.86, *P* = 0.024), low family support (hydration OR 0.58, 95% CI 0.44–0.76; sodium OR 0.29, 95% CI 0.12–0.70; both *P* ≤ 0.006), and frequent eating out (>3 times/week: hydration OR 0.63, 95% CI 0.47–0.84; sodium OR 0.34, 95% CI 0.14–0.85; both *P* ≤ 0.021) predicted lower adherence. Higher ambient temperature (>25 °C) increased hydration adherence (OR 1.67, 95% CI 1.30–2.14, *P* < 0.001).

**Table 6:** Environmental and occupational factors associated with adherence behaviors.

**Environmental factor**	**Prevalence**	**Hydration adherence**		**Sodium adherence**		***P* for**
	**No. (%)**	**OR (95% CI)**	***P*** **-value**	**OR (95% CI)**	***P*** **-value**	**Interaction** ^a^
**Workplace factors**						
Work shift pattern^b^						0.034
Day shift (reference)	1,098 (63.7)	1	–	1	–	–
Night shift	298 (17.3)	0.67 (0.51–0.88)	0.004	0.45 (0.19–1.07)	0.071	–
Rotating shifts	327 (19.0)	0.58 (0.44–0.76)	<0.001	0.38 (0.15–0.94)	0.037	–
Water availability at work						0.089
Always available	892 (51.8)	1	–	1	–	–
Usually available	534 (31.0)	0.78 (0.63–0.96)	0.021	0.71 (0.42–1.20)	0.201	–
Limited/never available	297 (17.2)	0.52 (0.40–0.68)	<0.001	0.34 (0.15–0.77)	0.01	–
Bathroom access at work						0.156
Unrestricted access	1,187 (68.9)	1	–	1	–	–
Restricted access	536 (31.1)	0.64 (0.52–0.79)	<0.001	0.58 (0.32–1.05)	0.072	–
Physical work intensity						0.023
Sedentary	621 (36.0)	1	–	1	–	–
Moderate	743 (43.1)	1.18 (0.95–1.46)	0.127	1.34 (0.77–2.33)	0.301	–
Heavy	359 (20.8)	1.89 (1.44–2.48)	<0.001	2.14 (1.15–3.98)	0.016	–
**Home environment**						
Water quality at home						0.241
Excellent/good	1,298 (75.3)	1	–	1	–	–
Fair/poor	425 (24.7)	0.71 (0.56–0.90)	0.005	0.89 (0.48–1.65)	0.713	–
Household cooking responsibility						0.007
Self/spouse primary cook	1,456 (84.5)	1	–	1	–	–
Others cook	267 (15.5)	0.87 (0.66–1.15)	0.329	0.31 (0.11–0.86)	0.024	–
**Climate and geographic factors**						
Average temperature (last month)^c^						0.412
<15 °C	431 (25.0)	1	–	1	–	–
15–25 °C	689 (40.0)	1.23 (0.97–1.56)	0.087	1.45 (0.78–2.69)	0.241	–
>25 °C	603 (35.0)	1.67 (1.30–2.14)	<0.001	1.89 (1.02–3.51)	0.043	–
Distance to healthcare facility						0.623
<30 min	1,289 (74.8)	1	–	1	–	–
≥30 min	434 (25.2)	0.78 (0.62–0.98)	0.032	0.67 (0.36–1.25)	0.208	–
**Social environment**						
Family support for diet changes						0.012
High support	743 (43.1)	1	–	1	–	–
Moderate support	687 (39.9)	0.82 (0.66–1.01)	0.064	0.61 (0.36–1.04)	0.067	–
Low/no support	293 (17.0)	0.58 (0.44–0.76)	<0.001	0.29 (0.12–0.70)	0.006	–
Peer influence (eating out frequency)						0.189
≤1 time per week	826 (47.9)	1	–	1	–	–
2–3 times per week	621 (36.0)	0.89 (0.72–1.10)	0.275	0.71 (0.42–1.20)	0.202	–
>3 times per week	276 (16.0)	0.63 (0.47–0.84)	0.002	0.34 (0.14–0.85)	0.021	–

CI, confidence interval; OR, odds ratio.

^a^*P*-value for interaction between environmental factor and urban vs. rural residence.

^b^Work shift patterns: day (7 a.m.−7 p.m.), night (7 p.m.−7 a.m.), rotating (alternating schedules).

^c^Temperature data obtained from local meteorological stations for month prior to interview. All ORs adjusted for age, sex, education, income, BMI, and stone history. Models include interaction terms where *P* < 0.05.

### 3.6 Knowledge-attitude-practice framework

Knowledge correlated moderately with adherence outcomes (*r* = 0.28–0.34), while self-efficacy showed stronger associations (*r* = 0.39–0.45; all *P* < 0.001; Table 7). Actual behaviors correlated highly with adherence classification (workday fluid intake *r* = 0.68; free-day intake *r* = 0.71; processed food avoidance *r* = 0.61). Structural equation modeling confirmed the knowledge → attitudes → behaviors → adherence pathway (CFI = 0.943, RMSEA = 0.056, SRMR = 0.048). Knowledge showed both direct (β = 0.18–0.32) and indirect effects (β = 0.06–0.12) mediated through attitudes and behaviors. Self-efficacy correlated positively with knowledge (*r* = 0.52) and behaviors (*r* = 0.38) while negatively with barriers (*r* = −0.41). The framework explained 28.9% of variance in hydration adherence, 34.1% in sodium adherence, and 38.7% in composite adherence.

**Table 7:** Correlation matrix and pathway analysis of knowledge-attitude-practice framework.

**KAP component**	**Correlation with adherence outcomes** ^**a**^			**Mediation analysis** ^**b**^		**Reliability**
	**Hydration**	**Sodium**	**Composite**	**Direct effect**	**Indirect effect**	**(Cronbach's** α**)**
	***r*** **(95% CI)**	***r*** **(95% CI)**	***r*** **(95% CI)**	β **(SE)**	β **(SE)**	
**Knowledge components**						
Overall knowledge score (0–10)	0.34	0.28	0.31	0.18	0.12	0.82
	(0.29–0.39)	(0.23–0.33)	(0.26–0.36)	−0.04	−0.03	
Hydration guidelines knowledge	0.41	0.19	0.35	0.32	0.08	0.76
	(0.36–0.46)	(0.14–0.24)	(0.30–0.40)	−0.05	−0.03
Sodium guidelines knowledge	0.22	0.38	0.33	0.29	0.09	0.79
	(0.17–0.27)	(0.33–0.43)	(0.28–0.38)	−0.05	−0.03	
Risk factor awareness	0.26	0.24	0.27	0.15	0.11	0.74
	(0.21–0.31)	(0.19–0.29)	(0.22–0.32)	−0.04	−0.03	
**Attitude components (health belief model)**						
Perceived susceptibility (1–5)	0.18	0.16	0.19	0.11	0.06	0.71
	(0.13–0.23)	(0.11–0.21)	(0.14–0.24)	−0.04	−0.02	
Perceived severity (1–5)	0.14	0.13	0.15	0.08	0.05	0.69
	(0.09–0.19)	(0.08–0.18)	(0.10–0.20)	−0.04	−0.02	
Perceived benefits (1–5)	0.21	0.24	0.25	0.14	0.09	0.77
	(0.16–0.26)	(0.19–0.29)	(0.20–0.30)	−0.04	−0.03	
Self-efficacy (1–5)	0.42	0.39	0.45	0.31	0.13	0.84
	(0.37–0.47)	(0.34–0.44)	(0.40–0.50)	−0.05	−0.04	
**Practice/behavior components**						
Fluid intake (workday), L/d	0.68	0.12	0.51	0.58	0.09	–
	(0.65–0.71)	(0.07–0.17)	(0.47–0.55)	−0.06	−0.03	
Fluid intake (free day), L/d	0.71	0.15	0.54	0.62	0.08	–
	(0.68–0.74)	(0.10–0.20)	(0.50–0.58)	−0.06	−0.03	
Water bottle carrying frequency	0.56	0.18	0.43	0.47	0.08	0.73
	(0.52–0.60)	(0.13–0.23)	(0.38–0.48)	−0.05	−0.03	
Added salt usage (reverse scored)	0.11	0.54	0.38	0.48	0.06	0.71
	(0.06–0.16)	(0.50–0.58)	(0.33–0.43)	−0.05	−0.02	
Processed food avoidance	0.18	0.61	0.45	0.53	0.07	0.76
	(0.13–0.23)	(0.57–0.65)	(0.40–0.50)	−0.06	−0.03	
Nutrition label reading	0.23	0.41	0.36	0.28	0.12	0.78
	(0.18–0.28)	(0.36–0.46)	(0.31–0.41)	−0.05	−0.04	
**Inter-component correlations** ^c^						
Knowledge ↔ perceived benefits	0.45	–	–	–	–	–
	(0.40–0.50)
Knowledge ↔ self-efficacy	0.52	–	–	–	–	–
	(0.47–0.57)
Self-efficacy ↔ behaviors	0.38	–	–	–	–	–
	(0.33–0.43)
Perceived barriers ↔ Self-efficacy	−0.41	–	–	–	–	–
	(−0.46 to −0.36)
**Model fit statistics** ^d^						
Comparative Fit Index (CFI)	0.943	–	–	–	–	–
Root Mean Square Error (RMSEA)	0.056	–	–	–	–	–
Standardized root mean residual	0.048	–	–	–	–	–
Total variance explained (*R*^2^)	0.289	0.341	0.387	–	–	–

CI, confidence interval; SE, standard error.

^a^Pearson correlation coefficients with 95% confidence intervals calculated using Fisher's z-transformation. All correlations *P* < 0.001 unless otherwise noted.

^b^Structural equation modeling with knowledge → attitudes → behaviors → adherence pathway. Direct effects control for mediating variables; indirect effects represent mediated pathways.

^c^Key theoretical relationships in the Knowledge-Attitude-Practice framework.

^d^Structural equation model fit indices for the complete KAP framework. CFI ≥0.95, RMSEA ≤0.06, and SRMR ≤0.08 indicate acceptable fit. Bootstrap confidence intervals (*n* = 5,000) used for mediation analysis. All models adjusted for demographics and clinical factors.

### 3.7 Stone recurrence and prevention effects

Among 1,668 participants with 12-month follow-up (96.8% retention), 312 experienced symptomatic recurrence (18.1%; Table 8). Previous stone episodes (OR 1.34 per episode, 95% CI 1.21–1.49, *P* < 0.001; PAF 45.2%), male sex (OR 1.45, 95% CI 1.02–2.06, *P* = 0.039; PAF 22.8%), and BMI (OR 1.08 per kg/m^2^, 95% CI 1.03–1.13, *P* = 0.002; PAF 31.4%) independently predicted recurrence. Hydration adherence was associated with lower recurrence risk (OR 0.68, 95% CI 0.48–0.96, *P* = 0.029), as was sodium adherence (OR 0.31, 95% CI 0.12–0.79, *P* = 0.014). Among biomarkers, higher urine volume (OR 0.54 per liter, 95% CI 0.38–0.76, *P* < 0.001; PAF 18.7%) and citrate (OR 0.93 per 100 mg/day, 95% CI 0.87–0.99, *P* = 0.028; PAF 8.4%) reduced risk, while higher urine sodium (OR 1.42 per 50 mmol/day, 95% CI 1.18–1.71, *P* < 0.001; PAF 26.5%) and calcium (OR 1.15 per 50 mg/day, 95% CI 1.05–1.26, *P* = 0.003; PAF 12.8%) increased risk. Ambient temperature (OR 1.19 per 5 °C, 95% CI 1.04–1.37, *P* = 0.012; PAF 7.3%) independently predicted recurrence. Model C-statistic was 0.723 (95% CI 0.684–0.762) with good calibration (*P* = 0.618).

**Table 8:** Clinical and behavioral predictors of 12–months stone recurrence.

**Predictor variable**	**Univariable**		**Multivariable**		**PAF (%)^a^**
	**OR (95% CI)**	***P*** **-value**	**OR (95% CI)**	***P*** **-value**	
**Demographics and clinical factors**					
Age (per year)	1.00 (0.99–1.02)	0.523	1.01 (0.99–1.03)	0.234	–
Male sex	1.52 (1.08–2.13)	0.016	1.45 (1.02–2.06)	0.039	22.8
BMI (per kg/m^2^)	1.09 (1.04–1.14)	<0.001	1.08 (1.03–1.13)	0.002	31.4
Urban residence	0.89 (0.64–1.25)	0.507	0.92 (0.65–1.30)	0.63	–
University education	0.78 (0.56–1.09)	0.143	0.83 (0.58–1.18)	0.299	–
Hypertension	1.67 (1.19–2.34)	0.003	1.42 (0.99–2.03)	0.056	–
Diabetes	1.89 (1.18–3.03)	0.008	1.58 (0.96–2.60)	0.071	–
Previous stone episodes (per episode)	1.38 (1.25–1.53)	<0.001	1.34 (1.21–1.49)	<0.001	45.2
**Adherence behaviors**					
Hydration adherent	0.62 (0.44–0.87)	0.006	0.68 (0.48–0.96)	0.029	15.3
Sodium adherent	0.28 (0.11–0.72)	0.008	0.31 (0.12–0.79)	0.014	2.1
Composite adherent	0.15 (0.02–1.11)	0.064	0.18 (0.02–1.35)	0.095	–
**Knowledge, attitudes, and practices**					
Knowledge score (per point)	0.91 (0.85–0.98)	0.012	0.93 (0.86–1.00)	0.058	–
Self-efficacy (per point)	0.83 (0.72–0.96)	0.014	0.87 (0.75–1.01)	0.071	–
Prior counseling received	0.71 (0.50–1.00)	0.049	0.76 (0.53–1.09)	0.138	–
**24-h urine parameters** ^b^					
Urine volume (per L)	0.51 (0.36–0.72)	<0.001	0.54 (0.38–0.76)	<0.001	18.7
Urine sodium (per 50 mmol/d)	1.48 (1.23–1.78)	<0.001	1.42 (1.18–1.71)	<0.001	26.5
Urine calcium (per 50 mg/d)	1.18 (1.08–1.29)	<0.001	1.15 (1.05–1.26)	0.003	12.8
Urine citrate (per 100 mg/d)	0.91 (0.85–0.97)	0.005	0.93 (0.87–0.99)	0.028	8.4
**Environmental factors**					
Average temperature (per 5 °C)	1.23 (1.08–1.41)	0.003	1.19 (1.04–1.37)	0.012	7.3
Limited water access at work	1.58 (1.12–2.23)	0.009	1.41 (0.98–2.03)	0.063	–
**Model performance**					
Nagelkerke *R*^2^	–	–	0.156	–	–
C-statistic (95% CI)	–	–	0.723 (0.684–0.762)	–	–
Hosmer–Lemeshow *P*-value	–	–	0.618	–	–

CI, confidence interval; OR, odds ratio; PAF, population attributable fraction.

^a^Population attributable fractions assume causal relationships and complete risk factor elimination, neither established from observational data. These represent theoretical upper bounds, not realistic intervention targets. PAFs are susceptible to residual confounding, measurement error, and unmeasured common causes. Observational PAFs may overestimate true intervention effects, as real-world adherence improvements are partial, and intervention effects may be smaller than observed associations due to self-selection and confounding by indication. These estimates are hypothesis-generating, not bases for quantitative intervention planning.

^b^24-h urine parameters available for 872 participants. Missing data imputed using multiple imputations for full cohort analysis. Stone recurrence is defined as symptomatic episode requiring medical attention or imaging confirmation within 12 months of enrollment. Follow-up rate: 96.8% (*n* = 1,668).

## 4 Discussion

This study addresses a critical implementation gap by integrating biomarker-validated adherence measures, psychosocial determinants within a KAP framework, and environmental and occupational exposures, and by linking these domains to 12-month stone recurrence. In contrast to prior observational work that relied largely on unvalidated self-report or limited biomarker panels, the present analysis applies 24-h urine chemistries to calibrate adherence classifications and thereby strengthens causal inference concerning modifiable behaviors. These findings clarify where the prevention cascade fails—particularly sodium restriction—and indicate practical leverage points for secondary prevention in real-world settings.

The baseline profile demonstrates a striking discordance between hydration adherence (56.5%) and sodium adherence (3.0%), despite moderate knowledge, high perceived severity, and only average self-efficacy. This pattern is consistent with clinical guidelines emphasizing high urine volume (≥2.0–2.5 L/day) and dietary sodium moderation as foundational elements of prevention, yet it underscores the well-documented difficulty of sustaining these behaviors outside controlled trials (31, 35). The mean urinary sodium excretion of 178.4 mmol/day—corresponding to approximately 10 g/day salt intake—exceeds recommended targets and aligns with mechanistic evidence linking sodium intake to calciuria and increased supersaturation of calcium salts (31, 35–37). Notably, nearly two in five participants reported prior dietary counseling; nevertheless, sodium adherence remained minimal, suggesting that didactic education without structured behavioral support and environmental enablement is inadequate. In contrast, the comparatively higher hydration adherence likely reflects a lower cognitive and logistical burden than sodium reduction, consonant with reports that cueing, access to water, and reminders are often sufficient to increase urine volume in motivated patients (38–40). Collectively, these observations reveal a large, actionable implementation gap for sodium reduction within guideline-based care.

The criterion validity of self-reported adherence against biomarkers is a central strength and a key advance relative to previous literature. Self-reported hydration adherence exhibited high sensitivity and specificity for the ≥2.5 L/day urine volume threshold, while sodium-adherent participants demonstrated markedly lower urinary sodium. These gradients are concordant with dose–response relationships between 24-h urinary chemistries and stone risk reported in contemporary analyses, which show that higher urine volume and citrate are protective and that higher urinary sodium and calcium increase risk (36, 41). Furthermore, randomized evidence in hypercalciuric stone formers demonstrates that normal calcium intake combined with reduced sodium and animal protein reduces recurrence more effectively than low-calcium diets, thereby supporting sodium moderation as a principal therapeutic target (37, 42). In contrast to earlier studies that dichotomized urinary thresholds, the present data, together with modern risk-prediction work, indicate that incremental behavioral improvements confer measurable reductions in risk, which justifies stepwise, individualized targets in clinical counseling (5, 42, 43).

Gradients across knowledge, perceived susceptibility and benefits, and self-efficacy by urban residence and educational attainment parallel findings from recent Chinese KAP studies that documented adequate or moderate knowledge but suboptimal practices and that confirmed a positive path from knowledge to attitudes and onward to behaviors (22, 44). The predominance of taste and environmental barriers in the present cohort is consistent with reports that forgetting to drink, poor water access, limited control over food procurement, and hedonic preferences impede adherence (38–40). These convergent data suggest that effective programs require coupling education with behavior-change techniques (e.g., goal-setting, action planning, habit formation) and with structural enablers (e.g., cueing technologies, improved workplace water and bathroom access, and family-level strategies to reshape the home food environment). Importantly, the observation that prior counseling, knowledge, and self-efficacy independently predicted adherence indicates that capabilities and motivation are necessary but not sufficient; affordability and access constraints must also be addressed to translate intention into behavior.

The multivariable models underscore this dual imperative. Prior dietetic counseling was the strongest predictor across all adherence outcomes, with additional independent effects of knowledge and self-efficacy, thereby corroborating evidence that multicomponent behavioral interventions—particularly those employing digital prompts, adaptive incentives, and structured problem-solving—can sustain fluid intake and improve urinary volume (38–40, 45). Simultaneously, access and affordability barriers exerted significant adverse effects on hydration, sodium, and composite adherence, aligning with implementation science that emphasizes environmental restructuring and price signals as determinants of sustained dietary change (65–67). The protective associations of urban residence and university education likely reflect differences in resource availability, health literacy, and choice architecture; nevertheless, equity-oriented interventions will need to focus on rural settings and lower-education groups in whom barrier burdens were greatest (45, 46).

Environmental and occupational determinants emerged as salient correlations of adherence. Night-shift and rotating-shift work were associated with reduced hydration adherence, and limited workplace water and bathroom access exerted strong adverse effects. These observations are biologically plausible given the influence of circadian disruption on renal handling of water and electrolytes, as well as on sleep, fatigue, and cue-based behaviors (47, 48, 68, 69). Importantly, external evidence now indicates that shift work is associated with a higher risk of clinically recognized kidney stone events, with an accompanying editorial emphasizing the likely role of hydration constraints and lifestyle mediators in this association (47, 49, 50). The independent contribution of ambient temperature to recurrence in this cohort dovetails with a robust literature linking short-term heat exposure to increased stone presentations and projecting a growing climate-related burden (2, 51–53). In contrast, heavy physical work was associated with greater adherence to hydration and sodium targets in this study, plausibly reflecting thirst-driven intake and worksite water provision; however, net risk mitigation will depend on the balance between increased fluid intake, sweat-related losses, and sodium exposure, which suggests that occupation-specific prevention packages should integrate thermal ergonomics with dietetic counseling.

The structural equation modeling provides mechanistic clarity by confirming the pathway knowledge → attitudes → behaviors → adherence, with both direct and indirect effects of knowledge and stronger contributions from self-efficacy. This pattern is congruent with recent KAP analyses employing similar methods in both patients and the general population (22, 44). The relatively large, explained variance for composite adherence indicates that psychosocial determinants, while central, account for only part of the adherence phenotype; thus, complementary action on structural barriers remains essential. These findings offer a blueprint for stepped, context-aware programs: strengthen knowledge to recalibrate perceived susceptibility and benefits; train self-efficacy, self-monitoring, and habit formation; and deploy environmental and digital supports to consolidate behaviors in daily life.

The prospective outcomes further reinforce these implications. A 12-month symptomatic recurrence proportion of 18.1% underscores the early hazard after an index episode, consistent with epidemiological evidence showing substantial recurrence accumulation over subsequent years (52, 54). Protective associations for hydration adherence and higher urinary citrate, and adverse associations for urinary sodium and calcium, are directionally and mechanistically consistent with contemporary cohort analyses and randomized trials (39, 44, 55, 56). The sodium signal is particularly compelling given persistent baseline excess in intake; coupled with randomized data in hypercalciuric stone formers (4) and guideline recommendations (1, 2), these data support prioritizing sodium reduction as a high-yield target. The independent effect of ambient temperature on recurrence emphasizes the need for seasonally responsive prevention (e.g., dynamic fluid goals, workplace cooling strategies, and ready access to water), with a specific focus on shift workers and outdoor laborers who experience combined heat and access constraints (39, 57, 58). The observation that previous stone episodes and higher BMI independently predicted recurrence accords with meta-analytic evidence, suggesting that integrating weight management and metabolic risk reduction with stone-specific counseling may yield additive benefits (52, 54, 55). Recent evidence demonstrates that optimal cardiovascular health (Life's Essential 8 score) is inversely associated with stone risk further supports incorporating lithiasis prevention into broader cardiometabolic interventions (59).

Taken together, these results address several research gaps. First, few prior studies have validated self-reported adherence against 24-h urine biomarkers at scale while simultaneously modeling psychosocial and structural determinants; the present analysis demonstrates that brief, structured self-report can track biomarker-defined targets with high accuracy, thereby enabling pragmatic monitoring in clinical programs (58, 60, 61, 70). Second, although education has been linked to improved knowledge, there has been limited quantification of the relative contributions of knowledge vs. self-efficacy and of structural barriers; the multivariable estimates here indicate that capabilities and motivation must be paired with environmental enablement to achieve composite adherence. Third, real-world occupational and environmental exposures—including shift schedules, workplace water and bathroom access, and ambient temperature—are rarely incorporated into prevention models; the current data demonstrate that these exposures materially shape adherence and recurrence, thereby justifying integration of occupational health principles and climate adaptation into nephrolithiasis prevention (52, 61, 62). Finally, by prospectively linking adherence and biomarker profiles to recurrence with adequate model discrimination and calibration, the study provides implementation-level evidence that adherence-first strategies anchored in 24-h urine targets can reduce near-term recurrence risk.

These observational findings provide preliminary support for potential practice implications requiring confirmation in randomized controlled trials before implementation. If validated, routine integration of 24 h urine testing to set personalized goals for volume, sodium, and citrate could be coupled with multicomponent behavioral supports (action planning, digital cueing, structured problem solving) and structural enablers (workplace water and bathroom access, affordable lower sodium options) (31, 35, 36). Given the sodium adherence deficit, programs might emphasize gradual sodium titration, specifically targeting the reduction of high-salt condiments (e.g., soy sauce) inherent to Chinese cuisine, and greater cooking control. Occupational policies permitting regular hydration and bathroom breaks, plus heat aware counseling during warmer months, may benefit high risk workers; workplace interventions require evaluation in controlled trials (38, 40, 52). Embedding validated self-report tools alongside periodic 24-h urine monitoring might enable risk stratified outreach; addressing affordability barriers through vouchers or subsidized low sodium staples could improve equity, particularly for rural populations disadvantaged by lower health literacy and limited water infrastructure. These strategies require implementation research to establish effectiveness, acceptability, and sustainability.

This study exhibits several methodological strengths that advance the evidence base for kidney stone prevention. The integration of 24-h urine biomarker validation in over 50% of participants (*n* = 872) represents a critical advancement over prior adherence research, demonstrating high sensitivity and specificity of self-reported adherence against objective urinary volume and sodium thresholds. The large sample size (*n* = 1,723), exceptional 96.8% retention at 12-month follow-up, and linkage to prospective symptomatic recurrence outcomes enable robust clinical inference. The comprehensive multi-domain assessment addresses important literature gaps by incorporating validated KAP constructs alongside understudied environmental determinants (ambient temperature, home water quality) and occupational exposures (shift work, workplace water/bathroom access). Finally, the application of structural equation modeling to elucidate the knowledge → attitudes → behaviors → adherence pathway, combined with multivariable models adjusted for sociodemographic, clinical, and barrier domains, provides mechanistic insights that can inform tailored, context-aware intervention design.

Several limitations warrant consideration. The single-center, clinic-based design limits generalizability to community-dwelling stone formers, and lack of systematic refusal tracking introduces potential selection bias toward more motivated participants. Adherence was measured only at baseline; we did not reassess behaviors during 12-month follow-up, precluding analysis of behavioral maintenance or change trajectories. Baseline-adherent participants may have discontinued preventive practices; baseline non-adherent participants may have improved. Observed associations with recurrence reflect initial behavioral status rather than sustained adherence, limiting temporal precedence and causal inference. Only a single 24-h urine collection was obtained per participant. Individual sodium excretion varies by 30–50 mmol/day across different days; urine volume fluctuates with ambient temperature, exercise, and beverage choices. This temporal sampling introduces non-differential measurement error, likely biasing associations toward the null (regression dilution bias) and underestimating true effect sizes. Observed protective associations of higher urine volume and lower urinary sodium with recurrence may therefore represent conservative estimates. Misclassification near biomarker thresholds (2.5 L/day; 100 mmol/day) reduces apparent diagnostic accuracy of self-report measures. Operational adherence definitions combined guideline-based physiological targets with pragmatic behavioral indicators (≥4/5 frequency scales; ≤2 processed meals/week) lacking formal validation in prior studies. The sodium adherence model included 14+ predictors with only 51 events (EPV ≈3–4:1), below recommended 10–15:1 thresholds, increasing overfitting risk and producing potentially unstable estimates with inflated performance metrics (C-statistic 0.784). This model is exploratory, requiring replication in adequately powered samples. The biomarker validation sub-study enrolled 50.6% of participants (*n* =8 72/1,723) voluntarily; although analysis revealed no significant demographic differences between participants and non-participants, unmeasured selection bias cannot be ruled out. If volunteers were more adherent or motivated, validation statistics may be optimistically biased. Although multiple imputation addressed missing data under missing-at-random assumptions, unmeasured confounding from medication use (thiazides, citrate supplementation), dietary calcium intake, and occupation-specific hydration policies may bias effect estimates. Self-reported stone recurrence may underestimate asymptomatic formation or overestimate events in participants with heightened symptom awareness. The study was conducted in a specific geographic and cultural context with distinctive dietary patterns; findings may not generalize to settings with different food environments or healthcare systems. Finally, this observational design precludes causal inference regarding whether improving adherence through targeted interventions would yield similar recurrence reductions.

## 5 Conclusion

These results identify a major implementation gap in secondary prevention: adherence to sodium restriction is very low despite moderate knowledge and high perceived severity. Self-reported behaviors closely tracked 24-h urine chemistries; baseline adherence was observationally associated with 12-month recurrence, though causal relationships cannot be established from this cross-sectional design with prospective follow-up. Observed associations may be confounded by unmeasured factors and do not establish that improving adherence through interventions would yield similar risk reductions. Adherence reflects both individual capability (knowledge, self-efficacy) and multi-level constraints (shift work, limited workplace water/bathroom access, out-of-home food environments, affordability, heat), explaining why counseling—though strongly associated with adherence—remains insufficient at scale. These findings provide preliminary support for integrated programs pairing personalized biomarker targets with multicomponent behavioral support and structural enablement (hydration-supportive workplace policies, affordable lower-sodium options, climate-adaptive strategies), particularly for rural and lower-education groups and high-exposure occupations. Randomized controlled trials are essential to establish efficacy, cost-effectiveness, and implementation feasibility before definitive policy recommendations or widespread implementation.

## Data Availability

The raw data supporting the conclusions of this article will be made available by the authors, without undue reservation.

## References

[1] GBD 2021 Urolithiasis Collaborators. The global, regional, and national burden of urolithiasis in 204 countries and territories, 2000-2021: a systematic analysis for the Global Burden of Disease Study 2021. EClinicalMedicine. (2024) 78:102924. doi: 10.1016/j.eclinm.2024.102924 39640943 PMC11618031

[2] Stamatelou K Goldfarb DS. Epidemiology of kidney stones. Healthcare (Basel, Switzerland). (2023) 11:424. doi: 10.3390/healthcare11030424 36766999 PMC9914194

[3] Peerapen P Thongboonkerd V. Kidney stone prevention. Adv Nutr (Bethesda, Md). (2023) 14:555–69. doi: 10.1016/j.advnut.2023.03.002 36906146 PMC10201681

[4] Basiri A Tahvildari A Naji M Ziaeefar P Kashi AH. Determination of the kidney stone composition using infrared spectroscopy in Iran at a national referral center during 2019-2023. Asian J Urol. (2025) 12:72–8. doi: 10.1016/j.ajur.2024.07.004 39990070 PMC11840317

[5] Prezioso D Strazzullo P Lotti T Bianchi G Borghi L Caione P . Dietary treatment of urinary risk factors for renal stone formation. A review of CLU Working Group. Arch Italiano Urol Androl. (2015) 87:105–20. doi: 10.4081/aiua.2015.2.105 26150027

[6] Cheungpasitporn W Rossetti S Friend K Erickson SB Lieske JC. Treatment effect, adherence, and safety of high fluid intake for the prevention of incident and recurrent kidney stones: a systematic review and meta-analysis. J Nephrol. (2016) 29:211–9. doi: 10.1007/s40620-015-0210-4 26022722 PMC4831051

[7] Travers S Prot-Bertoye C Daudon M Courbebaisse M Baron S. How to monitor hydration status and urine dilution in patients with nephrolithiasis. Nutrients. (2023) 15:1642. doi: 10.3390/nu15071642 37049482 PMC10097240

[8] Sorokin I Pearle MS. Medical therapy for nephrolithiasis: State of the art. Asian J Urol. (2018) 5:243–55. doi: 10.1016/j.ajur.2018.08.005 30364650 PMC6197179

[9] Scales CD Jr., Desai AC, Harper JD, Lai HH, Maalouf NM, Reese PP, et al. Prevention of urinary stones with hydration (PUSH): design and rationale of a clinical trial. Am J Kidney Dis. (2021) 77:898-906.e1. doi: 10.1053/j.ajkd.2020.09.016 PMC8124076 33212205

[10] Tan M He FJ Wang C MacGregor GA. Twenty-four-hour urinary sodium and potassium excretion in china: a systematic review and meta-analysis. J Am Heart Assoc. (2019) 8:e012923. doi: 10.1161/JAHA.119.012923 31295409 PMC6662145

[11] Cupisti A Giannese D D'Alessandro C Benedetti A Panichi V Alfieri C . Kidney stone prevention: is there a role for complementary and alternative medicine? Nutrients. (2023) 15:877. doi: 10.3390/nu15040877 36839235 PMC9959749

[12] Seeger H Kaelin A Ferraro PM Weber D Jaeger P Ambuehl P . Changes in urinary risk profile after short-term low sodium and low calcium diet in recurrent Swiss kidney stone formers. BMC Nephrol. (2017) 18:349. doi: 10.1186/s12882-017-0755-7 29202723 PMC5715611

[13] Zeng G Mai Z Xia S Wang Z Zhang K Wang L . Prevalence of kidney stones in China: an ultrasonography based cross-sectional study. BJU Int. (2017) 120:109–16. doi: 10.1111/bju.13828 28236332

[14] Wang Q Wang Y Yang C Wang J Shi Y Wang H . Trends of urolithiasis in China: a national study based on hospitalized patients from 2013 to 2018. Kidney Dis (Basel, Switzerland). (2023) 9:49–57. doi: 10.1159/000527967 36756086 PMC9900463

[15] Sun H Wang X Zhang X Wang L Tao M Wang Y . High ambient temperature increases the number of emergency visits for upper urolithiasis in Hefei City, China. Heliyon. (2023) 9:e12856. doi: 10.1016/j.heliyon.2023.e12856 36711317 PMC9876836

[16] Zhou L Chen R He C Liu C Lei J Zhu Y . Ambient heat stress and urolithiasis attacks in China: implication for climate change. Environ Res. (2023) 217:114850. doi: 10.1016/j.envres.2022.114850 36427640

[17] Han B Li C Zhou Y Zhang M Zhao Y Zhao T . Association of salt-reduction knowledge and behaviors and salt intake in chinese population. Front Public Health. (2022) 10:872299. doi: 10.3389/fpubh.2022.872299 35509508 PMC9058069

[18] Fang K He Y Fang Y Lian Y. Dietary sodium intake and food sources among chinese adults: data from the CNNHS 2010-2012. Nutrients. (2020) 12:453. doi: 10.3390/nu12020453 32054013 PMC7071264

[19] Du W Wang H Zhang J Zhang X Wei N Li Y . Sodium content of restaurant dishes in China: a cross-sectional survey. Nutr J. (2022) 21:10. doi: 10.1186/s12937-022-00762-4 35177072 PMC8851779

[20] Shen Y Jiang L Yu J Chen B Liu A Guo Y. The burden of chronic kidney disease attributable to high sodium intake: a longitudinal study in 1990-2019 in China. Front Nutr. (2024) 11:1531358. doi: 10.3389/fnut.2024.1531358 39897530 PMC11783680

[21] Wang Y Liu S Zhao Q Wang N Liu X Zhang T . Analysis of dietary patterns associated with kidney stone disease based on data-driven approaches: a case-control study in Shanghai. Nutrients. (2024) 16:214. doi: 10.3390/nu16020214 38257107 PMC10818537

[22] Chen T Jiang Y Zhang P Wang F Chen B Yu D. Knowledge, attitude, and practice regarding stone formation and recurrence among urolithiasis patients: a cross-sectional study. Sci Rep. (2024) 14:28408. doi: 10.1038/s41598-024-80078-x 39557990 PMC11574188

[23] Skolarikos A Somani B Neisius A Jung H Petrík A Tailly T . Metabolic evaluation and recurrence prevention for urinary stone patients: an EAU guidelines update. Eur Urol. (2024) 86:343–63. doi: 10.1016/j.eururo.2024.05.029 39069389

[24] Perrier ET Armstrong LE Bottin JH Clark WF Dolci A Guelinckx I . Hydration for health hypothesis: a narrative review of supporting evidence. Eur J Nutr. (2021) 60:1167–80. doi: 10.1007/s00394-020-02296-z 32632658 PMC7987589

[25] Kern A Grimsby G Mayo H Baker LA. Medical and dietary interventions for preventing recurrent urinary stones in children. Cochrane Database Syst Rev. (2017) 11:Cd011252. doi: 10.1002/14651858.CD011252.pub2 29117629 PMC6486163

[26] Tariq MH Sulaiman SAS Farrukh MJ Goh KW Ming LC. Development and validation of Chronic Kidney Disease Knowledge, Attitude, and Practices (CKD-KAP) questionnaire. Front Med. (2022) 9:956449. doi: 10.3389/fmed.2022.956449 36304188 PMC9592727

[27] Song K Ye S Song J Kang Z. Knowledge attitude and practice of patients with allergic conjunctivitis towards their disease. Sci Rep. (2025) 15:6238. doi: 10.1038/s41598-025-87518-2 39979330 PMC11842743

[28] Zhang X Sun M Xue G Zhao Y Du T Guan X . Knowledge, attitudes, and practices of epilepsy patients regarding the ketogenic diet therapy: a cross-sectional study. Epilepsia Open. (2025) 10:866–79. doi: 10.1002/epi4.70048 40323735 PMC12163517

[29] World Health Organization (WHO). Guideline: Sodium Intake for Adults and Children 2012 09-12-2025. Available online at: https://www.who.int/publications/i/item/9789241504836 (Accessed December 10, 2025). 23658998

[30] Krauss RM Eckel RH Howard B Appel LJ Daniels SR Deckelbaum RJ . AHA dietary guidelines: revision 2000: a statement for healthcare professionals from the Nutrition Committee of the American Heart Association. Circulation. (2000) 102:2284–99. doi: 10.1161/01.CIR.102.18.2284 11056107

[31] Pearle MS Goldfarb DS Assimos DG Curhan G Denu-Ciocca CJ Matlaga BR . Medical management of kidney stones: AUA guideline. J Urol. (2014) 192:316–24. doi: 10.1016/j.juro.2014.05.006 24857648

[32] Türk C Petrík A Sarica K Seitz C Skolarikos A Straub M . EAU guidelines on interventional treatment for urolithiasis. Eur Urol. (2016) 69:475–82. doi: 10.1016/j.eururo.2015.07.041 26344917

[33] Cogswell ME Maalouf J Elliott P Loria CM Patel S Bowman BA. Use of urine biomarkers to assess sodium intake: challenges and opportunities. Annu Rev Nutr. (2015) 35:349–87. doi: 10.1146/annurev-nutr-071714-034322 25974702 PMC5497310

[34] Park SM Jee J Joung JY Cho YY Sohn SY Jin SM . High dietary sodium intake assessed by 24-hour urine specimen increase urinary calcium excretion and bone resorption marker. J Bone Metab. (2014) 21:189–94. doi: 10.11005/jbm.2014.21.3.189 25247156 PMC4170081

[35] Akram M Jahrreiss V Skolarikos A Geraghty R Tzelves L Emilliani E . Urological guidelines for kidney stones: overview and comprehensive update. J Clin Med. (2024) 13. doi: 10.3390/jcm13041114 38398427 PMC10889283

[36] Ferraro PM Taylor EN Curhan GC. 24-Hour urinary chemistries and kidney stone risk. Am J Kidney Dis. (2024) 84:164–9. doi: 10.1053/j.ajkd.2024.02.010 38583757 PMC13170619

[37] Borghi L Schianchi T Meschi T Guerra A Allegri F Maggiore U . Comparison of two diets for the prevention of recurrent stones in idiopathic hypercalciuria. N Engl J Med. (2002) 346:77–84. doi: 10.1056/NEJMoa010369 11784873

[38] Conroy DE Marks J Cutshaw A Ram N Thomaz E Streeper NM. Promoting fluid intake to increase urine volume for kidney stone prevention: protocol for a randomized controlled efficacy trial of the sip(IT) intervention. Contemp Clin Trials. (2024) 138:107454. doi: 10.1016/j.cct.2024.107454 38253254 PMC10923155

[39] Stout TE Lingeman JE Krambeck AE Humphreys MR Zisman A Elfering S . A randomized trial evaluating the use of a smart water bottle to increase fluid intake in stone formers. J Renal Nutr. (2022) 32:389–95. doi: 10.1053/j.jrn.2021.07.007 35283036

[40] Wessells H Lieske JC Lai HH Al-Khalidi HR Desai AC Harper JD . Adjudication of self-reported symptomatic stone recurrence in the prevention of urinary stones with hydration trial. Urology. (2024) 194:27–35. doi: 10.1016/j.urology.2024.08.026 39242045 PMC11625008

[41] Heilberg IP Goldfarb DS. Optimum nutrition for kidney stone disease. Adv Chronic Kidney Dis. (2013) 20:165–74. doi: 10.1053/j.ackd.2012.12.001 23439376

[42] Zisman AL. Effectiveness of treatment modalities on kidney stone recurrence. Clin J Am Soc Nephrol. (2017) 12:1699–708. doi: 10.2215/CJN.11201016 28830863 PMC5628726

[43] Ene MA Geavlete PA Simeanu CE Bulai CA Ene CV Geavlete BF. The effectiveness of citrates and pyridoxine in the treatment of kidney stones. J Med Life. (2023) 16:856–61. doi: 10.25122/jml-2023-0234 37675156 PMC10478649

[44] Lv D Tang L Chen Y Wang R Liu L Jian N . Knowledge, attitudes, and practices towards urinary system stones among the Chengdu population. Sci Rep. (2024) 14:11303. doi: 10.1038/s41598-024-60227-y 38760386 PMC11101414

[45] Reese PP Shah S Funsten E Amaral S Audrain-McGovern J Koepsell K . Using structured problem solving to promote fluid consumption in the prevention of urinary stones with hydration (PUSH) trial. BMC Nephrol. (2024) 25:183. doi: 10.1186/s12882-024-03605-y 38807063 PMC11134957

[46] Kühn L Bachert P Hildebrand C Kunkel J Reitermayer J Wäsche H . Health literacy among university students: a systematic review of cross-sectional studies. Front Public Health. (2021) 9:680999. doi: 10.3389/fpubh.2021.680999 35127605 PMC8814326

[47] Knauf F Luft FC Nath KA. Shift work and the risk of kidney stones. Mayo Clinic Proc. (2025). doi: 10.1016/j.mayocp.2025.08.018 41031993

[48] He SK Wang JH Li T Yin S Cui JW Xiao YF . Sleep and circadian rhythm disturbance in kidney stone disease: a narrative review. Front Endocrinol. (2023) 14:1293685. doi: 10.3389/fendo.2023.1293685 38089624 PMC10711275

[49] Sasai F Roncal-Jimenez C Rogers K Sato Y Brown JM Glaser J . Climate change and nephrology. Nephrol Dial Transplant. (2023) 38:41–8. doi: 10.1093/ndt/gfab258 34473287 PMC9869860

[50] Gauhar V Castellani D Tsaturyan A Taguchi K Herrmann T Somani B . Does the existing evidence on flexible and navigable suction ureteral access sheath indicate a potential paradigm shift in the management of kidney and ureteral stones with flexible ureteroscopy? An overview from EAU endourology. Curr Opin Urol. (2025) 1:1333. doi: 10.1097/MOU.0000000000001333 40923118

[51] Spiardi R Goldfarb DS Tasian GE. Role of climate change in urologic health: kidney stone disease. Eur Urol Focus. (2023) 9:866–8. doi: 10.1016/j.euf.2023.10.001 37839975

[52] Kaufman J Vicedo-Cabrera AM Tam V Song L Coffel E Tasian G. The impact of heat on kidney stone presentations in South Carolina under two climate change scenarios. Sci Rep. (2022) 12:369. doi: 10.1038/s41598-021-04251-2 35013464 PMC8748744

[53] Yang C Li S Yang Y Huang C Li Y Tan C . Heatwave and upper urinary tract stones morbidity: effect modification by heatwave definitions, disease subtypes, and vulnerable populations. Urolithiasis. (2024) 52:134. doi: 10.1007/s00240-024-01619-7 39361149

[54] Wang K Ge J Han W Wang D Zhao Y Shen Y . Risk factors for kidney stone disease recurrence: a comprehensive meta-analysis. BMC Urol. (2022) 22:62. doi: 10.1186/s12894-022-01017-4 35439979 PMC9017041

[55] Islam AK Holt S Reisch J Nwariaku F Antonelli J Maalouf NM. What predicts recurrent kidney stone after parathyroidectomy in patients with primary hyperparathyroidism? J Am Coll Surg. (2020) 231:74–82. doi: 10.1016/j.jamcollsurg.2020.04.015 32330575

[56] Zomorodian A Moe OW. Citrate and calcium kidney stones. Clin Kidney J. (2025) 18:sfaf244. doi: 10.1093/ckj/sfaf244 40978115 PMC12445640

[57] Kamal W Azhar RA Hamri SB Alathal AH Alamri A Alzahrani T . The Saudi urological association guidelines on urolithiasis. Urol Ann. (2024) 16:1–27. doi: 10.4103/ua.ua_120_23 38415236 PMC10896325

[58] Ioannou LG Foster J Morris NB Piil JF Havenith G Mekjavic IB . Occupational heat strain in outdoor workers: a comprehensive review and meta-analysis. Temperature (Austin, Tex). (2022) 9:67–102. doi: 10.1080/23328940.2022.2030634 35655665 PMC9154804

[59] Yang Q Lin H Zhang X Tang H Huang J Luo N . Life's Essential 8 and kidney stones in US adults: mediating roles of HDL and insulin resistance. Minerva Urol Nephrol. (2025) 77:120–9. doi: 10.23736/S2724-6051.24.05774-4 39792352

[60] Zhao Q Zhang C Zhang W Zhang S Liu Q Guo Y. Applications and challenges of biomarker-based predictive models in proactive health management. Front Public Health. (2025) 13:1633487. doi: 10.3389/fpubh.2025.1633487 40900695 PMC12399543

[61] Rudroff T. Digital biomarkers and AI for remote monitoring of fatigue progression in neurological disorders: bridging mechanisms to clinical applications. Brain Sci. (2025) 15:533. doi: 10.3390/brainsci15050533 40426703 PMC12110069

[62] Yanase T Unno R Tokas T Gauhar V Sasaki Y Kawase K . AI-driven prediction of renal stone recurrence following ECIRS: a machine learning approach to postoperative risk stratification incorporating 24-hour urine data. J Clin Med. (2025) 14:4037. doi: 10.3390/jcm14124037 40565784 PMC12193965

[63] Saqib MAN Siddiqui S Qasim M Jamil MA Rafique I Awan UA . Effect of COVID-19 lockdown on patients with chronic diseases. Diabetes Metab Syndr. (2020) 14:1621–3. doi: 10.1016/j.dsx.2020.08.028 32889403 PMC7450263

[64] Haqqi A Awan UA Ali M Saqib MAN Ahmed H Afzal MS. COVID-19 and dengue virus coepidemics in Pakistan: a dangerous combination for an overburdened healthcare system. J Med Virol. (2021) 93:80–2. doi: 10.1002/jmv.26144 32510175 PMC7300443

[65] Awan UA Khattak AA. Has Pakistan failed to roll back HPV? Lancet Oncol. (2022) 23:e204. doi: 10.1016/S1470-2045(22)00141-3 35489348

[66] Awan UA Guo X Khattak AA Hassan U Khan S. Economic crises and cancer care in Pakistan-timely action saves lives. Lancet. (2024) 403:613–4. doi: 10.1016/S0140-6736(23)01380-6 38368005

[67] Awan UA Khattak AA Bai Q Khan S. Pakistan's transgender health disparities-a threat to HPV elimination? Lancet Reg Health Southeast Asia. (2024) 24:100351. doi: 10.1016/j.lansea.2024.100351 38756159 PMC11096673

[68] Khan MZ Hussain M Khan AA Hassan U Akhter N Hameed M . Frequency of non-diabetic renal disease in type 2 diabetes mellitus patients undergoing renal biopsy. J Ayub Med Coll Abbottabad. (2021) 33(Suppl. 1):S757–S762. 35077622

[69] Bashir S Hussain M Ali Khan A Hassan U Mushtaq KS Hameed M . Renal transplant pathology: demographic features and histopathological analysis of the causes of graft dysfunction. Int J Nephrol. (2020) 2020:7289701. doi: 10.1155/2020/7289701 33489373 PMC7787863

[70] Awan UA Bashir S Hassan U Khan SN Awan FM Jabbar A . HPV-driven breast carcinogenesis: associations with tumor severity, Ki67 expression and metastasis. Infect Agent Cancer. (2025) 20:55. doi: 10.1186/s13027-025-00668-w 40804747 PMC12345114

